# Emerging evidence of genotype–phenotype associations of developmental and epileptic encephalopathy due to KCNC2 mutation: Identification of novel R405G

**DOI:** 10.3389/fnmol.2022.950255

**Published:** 2022-08-25

**Authors:** Sumei Wang, Yejing Yu, Xu Wang, Xiaolong Deng, Jiehui Ma, Zhisheng Liu, Weiyue Gu, Dan Sun

**Affiliations:** ^1^Department of Pediatric Neurology, Wuhan Children’s Hospital, Tongji Medical College, Huazhong University of Science and Technology, Wuhan, China; ^2^Department of Neurology, Changchun Children’s Hospital, Changchun, China; ^3^Chigene (Beijing) Translational Medical Research Center Co. Ltd., Beijing, China

**Keywords:** developmental and epileptic encephalopathy, whole-exome sequencing, potassium channels, KCNC2, Kv3.2, R405G

## Abstract

Developmental and epileptic encephalopathies (DEEs) have high genetic heterogeneity, and DEE due to the potassium voltage-gated channel subfamily C member 2 (KCNC2) variant remains poorly understood, given the scarcity of related case studies. We report on two unrelated Chinese patients, an 11-year-old boy and a 5-year-old girl, diagnosed with global developmental delay (GDD), intellectual disability (ID), and focal impaired awareness seizure characterized by generalized spike and wave complexes on electroencephalogram (EEG) in the absence of significant brain lesions. Whole-exome sequencing (WES) and electrophysiological analysis were performed to detect genetic variants and evaluate functional changes of the mutant KCNC2, respectively. Importantly, we identified a novel gain-of-function KCNC2 variant, R405G, in both patients. Previously reported variants, V471L, R351K, T437A, and T437N, and novel R405G were found in multiple unrelated patients with DEE, showing consistent genotype–phenotype associations. These findings emphasize that the KCNC2 gene is causative for DEE and facilitates treatment and prognosis in patients with DEE due to KCNC2 mutations.

## Introduction

Developmental and epileptic encephalopathy (DEE) is a group of epilepsy syndromes characterized by severe seizures that are drug-resistant and significant developmental delay or regression (Scheffer et al., 2017). Most patients with DEE exhibit onset during infancy with different types of seizures, e.g., epileptic spasms, tonic, or atonic seizures, and myoclonic seizures. Over 50 causative genes have been found in DEE cases, indicating high heterogeneity in both phenotype and etiopathogenesis of DEE (He et al., 2019; Raga et al., 2021). Impairment of potassium ion subunits, e.g., potassium voltage-gated channel subfamily A, B, and Q (KCNA, KCNB, and KCNQ) members, is well-established to play an important role in DEE (Lal et al., 2020). In comparison with potassium voltage-gated channel subfamily C, member 1 (KCNC1) and member 3 (KCNC3), which were previously reported to be associated with DEE, progressive myoclonus epilepsy (MIM: #616187), and spinocerebellar ataxia (MIM: #605259) (Muona et al., 2015; Zhang and Kaczmarek, 2016), respectively, the linkage of the potassium voltage-gated channel subfamily C member 2 (*KCNC2*) gene and DEE remains poorly understood due to the scarcity of reported cases (Rademacher et al., 2020; Vetri et al., 2020; Rydzanicz et al., 2021; Schwarz et al., 2022).

The *KCNC2* gene, also known as K_*V*_3.2, together with *KCNC1*, *KCNC3*, and *KCNC4* (K_*V*_3.4), belongs to the Shaw-related potassium channel family (K_*V*_3 subfamily). It is well-established that K_*V*_3 proteins share the structure of six-transmembrane (6-TM) voltage-gated K + channels, which maintain the potassium ion permeability of excitable membranes by regulating pore penetration in 6-TM in response to voltage changes, thereby maintaining neuronal functions, such as maintenance of membrane potential, regulation of cell volume or neurotransmitter release, and modulation of electrical excitability (González et al., 2012; Kaczmarek and Zhang, 2017). In the human brain, K_*V*_3 channels act as potential regulators of neurons and rapidly activate at high-threshold voltages and deactivate very fast, suggesting a role in the excitability of the central nervous system (CNS) by regulating the membrane resting potential and fast action (Rudy et al., 1999; Rudy and McBain, 2001; McDonald and Mascagni, 2006).

The *KCNC2* expression is relatively high in the human brain, particularly in the hippocampus, frontal cortex, anterior cingulate cortex, caudate, hypothalamus, basal amygdala, and pituitary, playing a key role in regulating the excitability and neural communication (Chow et al., 1999). Recent human single-cell transcriptome sequencing (scRNA-seq) studies have demonstrated that KCNC2 is enriched in both inhibitory and excitatory neurons.^1^ An increasing body of evidence suggests that KCNC2 is essential for subserving a fast-spiking firing pattern and sustaining trains of action potentials at high frequencies to maintain an effective synaptic transmission and modulate the excitation (Erisir et al., 1999; Atzori et al., 2000). It has been shown that knockout of Kcnc2 (K_*V*_3.2^–/–^) in mice resulted in a deficit of high-frequency oscillations modulation, distorted cortical rhythmic activity, suppressed cortical inhibition, impaired fast spiking in cortical interneurons, enhanced susceptibility to epileptic seizures and abnormal sleep patterns, and increased anxiety in the open field. However, it can be challenging to discern whether a neurodevelopmental delay has occurred (Lau et al., 2000; Yu et al., 2006). In addition, *KCNC2* homolog-associated seizures are found in other animal models: delayed rectifier downregulated channels K_*V*_3.1-2 are observed in the hippocampus of adult gerbils during seizures (Lee et al., 2009), while overexpression of *KCNC2* in thalamocortical circuits is thought to be the cause of absence epilepsy (Stern et al., 2020). This *in vivo* evidence suggests that human *KCNC2* loci are linked to epilepsies.

In this study, we identified a novel heterozygous *KCNC2* variant, c.1213A > G (p. R405G), in two unrelated patients who were both clinically diagnosed with DEE. We demonstrated that the variant R405G yielded higher activity *in vitro* than the wild-type (WT) KCNC2 on a range of functioning voltages.

## Materials and methods

This study was approved by the Ethics Committee of Wuhan Children’s Hospital, Hubei, China (serial number: 2022497). Written informed consent for genetic testing and publication of photographs was obtained from the legal guardians of each subject. All investigations were conducted in accordance with the principles of the Declaration of Helsinki.

### Subjects

Two patients from unrelated families were admitted to the Department of Neurology, Wuhan Children’s Hospital. The medical records, including medical history, symptoms and signs, physical examinations, laboratory tests, radiological results, and developmental assessment, were documented by the attending physicians. Genetic tests and analyses were performed by Chigene (Beijing, China).

### Whole-exome sequencing

Whole-exome sequencing (WES) was performed on the proband’s genomic DNA extracted from peripheral blood leukocytes using xGen Exome Research Panel version 1.0 (IDT, IA, United States) and paired-end sequencing (2 × 150 bp) on NovaSeq 6000 (Illumina, San Diego, CA, United States) with not less than 99% regions covered at least 10×. Raw sequence readouts were processed by fastp. Paired-end sequence reads were aligned to the human GRCh37/hg19 genome with Burrows–Wheeler Aligner (BWA). Initial mapping process and variant site identification were conducted using GATK. Annotations were retrieved from the database-based online system independently developed by Chigene (Beijing, China),^2^ including allele frequency in the Single Nucleotide Polymorphism database (dbSNP), 1000 Genomes Project, Exome Aggregation Consortium (ExAC), Genome Aggregation Database (gnomAD), ESP, and Chigene in-house MAFs database; positions and associated clinical phenotypes based on UCSC, RefGene, GENCODE, and ENSEMBL transcripts; LOVD, SWISS, Clinvitae, HGMD, OMIM, and ClinVar databases; and functional and conservational predictions for the amino-acid changes according to software packages (Provean, SIFT, PolyPhen2, MutationTaster, M-CAP, REVEL, and CADD; MaxEntScan, dbscSNV, and GTAG; GERP, phyloP, and phastCons). Variant filtering was done according to rare variants with minor allele frequency (MAF) of <1% and biological impact, gene features and disease mechanism, recessive/dominant/*de novo* pattern of inheritance and segregation analysis, phenotypic relevance through human phenotype ontology (HPO) terms, and scientific literature. Subsequently, pathogenic or likely pathogenic variants were evaluated according to the American College of Medical Genetics (ACMG) practice guidelines.

### Sanger sequencing

The KCNC2 (NM_139137) variant c.1213A > G treated as plausible disease-causing was verified in the probands and their parents by Sanger sequencing performed using the BigDye Terminator version 1.1 Cycle Sequencing Kit on an ABI 3730XL instrument (Applied Biosystems, Waltham, MA, United States) to define the inheritance model. The primers used are 5′-CTGACCATGTTTGGGGGTACA-3′ and 5′-GAAACGGATCCTGCCTTGAC-3′ (product size of 520 bp and annealing temperature of 60°C). PCR amplification was carried out with a KAPA2G Robust HotStart PCR Kit (KAPA Biosystems, Roche, Basel, Switzerland) on a Hema 9600 PCR Thermo Cycler (Zhuhai Hema Medical Instrument Co., Ltd., Zhuhai, China). The DNA sequences were analyzed using the DNASTAR software package.

### Molecular modeling

Predicted (AF-Q96PR1-F1) configurations of human KCNC2 in the AlphaFold Protein Structure Database served as the template for developing a structural model that covered all 638 residues (NP_631875.1). Hydrogen-bond networks of the WT and the variant proteins were visualized in PyMOL.

### Cell culture and transfection

HEK293 cells were cultured at 37°C in DMEM supplemented with 10% fetal bovine serum (FBS) (AusGeneX, Oxenford, QLD, Australia) in a humidified 5% (v/v) CO_2_ atmosphere. HEK-293 cells were seeded in six-well plates and subsequently transfected with 2 μg of WT or mutant cDNAs by transient transfection using Lipofectamine 3000 (Invitrogen, Waltham, MA, United States). The day after transfection, cells were seeded into a 24-well plate at a density of 8 × 10^3^ cells per well. Approximately 48 h after transfection, cells were selected based on the intensity of the fluorescent reporter and placed in the recording chamber.

### Electrophysiological analysis

The macroscopic current of transiently transfected HEK-293 cells was recorded with an EPC10 amplifier (HEKA, Lambrecht, Germany) and the Patchmaster software using the whole-cell configuration of the patch-clamp technique. The sampling interval was set at 100 ms (20 kHz) in all experiments. Pipettes (Sutter Instruments, United States) were pulled from borosilicate glass and had resistances between 1.5 and 2.5 MΩ when filled with the pipette solution. The glass pipette was pulled using a micropipette puller and manipulated using a micro-manipulator under the microscope. After touching the cell, a slight suction was applied to achieve high seal resistance (GΩ). Fast capacitance (in pF) compensation was made after achieving a high seal, and the membrane was broken. Cell capacitance (in pF) compensation was made from whole-cell capacitance compensation after the whole-cell mode was achieved. No leak subtraction was made. The extracellular solution contained (in mM) 140 sodium chloride (NaCl), 3.5 potassium chloride (KCl), 1 MgCl_2_.6H_2_O, 2 CaCl_2_.2H_2_O, 10 D-glucose, 10 N-2-hydroxyethylpiperazine-N′-2-ethanesulfonic acid (HEPES), 1.25 NaH_2_PO_4_.2H2O, and pH adjusted to 7.4 with sodium hydroxide (NaOH). The pipette solution contained (in mM) 20 KCl, 115 K-aspartic, 1 MgCl_2_.6H_2_O, 5 ethylene glycol tetraacetic acid (EGTA), 10 HEPES, 2 Na_2_-ATP, and pH adjusted to 7.2 using potassium hydroxide (KOH). After the break-in, a 5-min waiting period equilibrated the cytoplasm of the cell and the pipette solution. Currents were activated by 500 ms voltage pulses ranging from −80 to +80 mV (in 10 mV increments) from a holding potential of −80 mV at a sweep frequency of 100 ms, calculated by dividing the current (in pA) by the cell capacitance (in pF). Current–voltage curves were converted through a Boltzmann equation (g(V) = gmax/(1 + exp((V-V0.5)/k))), where g, conductance; gmax, maximal conductance; V, voltage; V0.5, voltage of half-maximal activation; and k, slope factor into normalized conductance–voltage curve.

### Statistical analysis

Data are expressed as mean ± standard error of the mean (SEM). At each measured voltage, e.g., −40, +10, and +60 mV, the membrane currents of mutant and WT cells were repeatedly tested three times, taking the mean value and SEM for *t*-test comparison. Significant differences were assessed by Student’s *t*-test, with *p < 0.05, ^**^P < 0.01, ^***^P < 0.001, and ^****^P < 0.0001.

### Literature search

Keywords “KCNC2” or “K_*V*_3.2,” “developmental and epileptic encephalopathy,” “DEE,” or “epilepsy” were used for literature search in the PubMed, Medline, and Google Scholar databases.

## Results

### Case presentation

#### Patient 1

**FIGURE 1:**
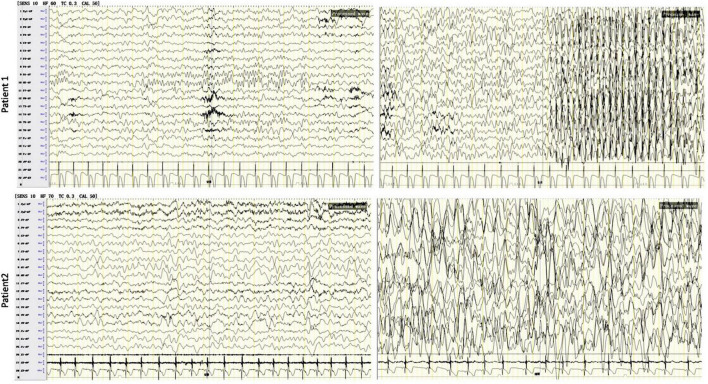
Electroencephalogram (EEG) tests in patients with developmental and epileptic encephalopathie (DEE) due to the KCNC2 R405G variant. The results showed background **(left)** generalized, diffuse spike, and wave complexes in both patients. In patient 1 (at the age of 9 years), the EEG showed generalized high amplitude waves at 3–4 Hz and spike-and-slow-wave bursts for 8–18 s in an epileptic attack **(upper right)**. In patient 2, a sleep EEG **(lower right)** showed slowing of the occipital basic rhythm, multifocal generalized complexes of slow wave, spike and slow wave, sharp slow wave, and polyspike and slow wave, especially in occipital, frontal pole, and frontal regions.

Patient 1 was a 9-year-old boy admitted to our hospital for intractable epilepsy. He was born to a non-consanguineous Chinese couple with a history of birth asphyxia and hypoxia. The parents claimed no family history of seizures. At the age of 3 months, the patient experienced paroxysmal attacks with rolling back of eyeballs and loss of consciousness several times per day, which led to an initial diagnosis of focal epilepsy at the local hospital. For the following 8 years, he was successively treated with levetiracetam (LEV), valproate acid (VPA), oxcarbazepine (OXC), lamotrigine (LTG), and nitrazepam (NZP), which were ineffective for seizure control. Despite normal growth, the patient had intellectual disability (ID) with language and motor impairment. At the age of 9 years, the patient could not run and jump and had moderate ID (IQ 56). He experienced several focal seizures with episodic atypical absences during his hospitalization. Electroencephalogram (EEG) showed slowing of the basic background rhythm with hypsarrhythmia and generalized multifocal polyspike and slow wave, especially in the sleep stage. During a seizure, EEG showed generalized high amplitude waves at 3–4 Hz and spike-and-slow-wave bursts for 8–18 s. His blood and urine tests and brain MRI were normal. The patient was seizure-free after treatment with a combination of VPA/LTG/NZP. At the last follow-up, the patient (at the age of 11 years) was seizure-free with multifocal slow-wave, sharp-slow-wave, sharp-wave, and spike complexes on awake EEG. Complexes of multifocal polyspike and slow wave, spike and slow-wave, and sharp-wave were conducted by forward modeling EEG during sleep (Figure 1 and Table 1).

**TABLE 1:** Clinical features and KCNC2 variants in patients with developmental and epileptic encephalopathie (DEE) (*n* = 13).

Pat.	Sex	Age at last examination	Mut., inh.	Clinical diagnosis	Seizure types	EEG	Brain MRI	DD/ID	Anti-epilepsy treatment	References
1	Male	11 years	R405G, NA	DEE	Focal unaware, absence	Occipital basic rhythm slowing; multifocal slow-wave, sharp-slow-wave, sharp-wave, and spikes complexes in awake; multifocal polyspike-and-slow-wave, spike-and-slow-wave, and sharp-wave complexes, left forward in sleeping	Normal	Moderate to severe ID, not able to run and jump	SF (VPA/LTG/NZP)	Present study
2	Female	4 years	R405G, *de novo*	DEE	Focal unaware	Occipital basic rhythm slowing; multifocal generalized spikes, spike-and-slow-wave, polyspike- and-slow-wave, and sharp wave complexes	Normal, unsignificant nodular signal	Severe ID, no speech, ataxic gait, not able to run and jump	SF (VPA/LTG)	Present study
3	Female	7 years	D167Y, *de novo*	DEE: WS to LGS	IS, focal unaware, tonic-clonic, atypical Abs, Myo, SE, GTCS	Hypsarrhythmia, multifocal generalized spikes, missing background activity, sleep architecture	Arachnoidal cysts left temporo-basal, delayed myelination, progressive brain atrophy	Moderate to severe ID, ataxic gait, hypotonia, speech disturbance, macrocephaly	DR	Rademacher et al., 2020
4	Male	8 years	V471L, *de novo*	DEE	IS	Quasi-continuous burst-suppression pattern, generalized and high voltage spike-and-wave complexes	Widespread hypomyelination, hypertrophic frontal lobes	Severe ID, no speech, drooling, spastic tetraplegia, feeding difficulties	DR	Vetri et al., 2020
5	Female	2 years	V471L, *de novo*	DEE: WS	IS, polymorphic, tonic, tonic-clonic	Multifocal generalized spikes, polyspikes, spike-and-slow-wave, spike, and sharp wave fast complexes	Mild internal and external hydrocephalus	Severe ID, not able to sit, lack head control, hypotonia	Mild improved (lacosamide)	Rydzanicz et al., 2021
6	Male	1.5 ms	C125W, *de novo*	DEE: EOAE	Abs, Myo, GTCS	G(psw)	Normal	Mild - moderate	SF (VPA/CZP)	Schwarz et al., 2022
7	Male	1 year	E135G, *de novo*	DEE	Myo, atonic	G	Normal	Mild	DR	Schwarz et al., 2022
8	Male	7 ms	R351K, *de novo*	DEE: CSWS	GTCS, Myo, focal unaware	CSWS	Normal	Mild	SF (VPA)	Schwarz et al., 2022
9	Male	8 ms	R351K, *de novo*	DEE: CSWS	GTCS, Myo, Abs, focal unaware	CSWS	Normal	Severe	DR	Schwarz et al., 2022
10	Male	8 ms	T437A, *de novo*	DEE: EOAE	Abs, tonic	G	Normal	Severe	DR	Schwarz et al., 2022
11	Female	11 ms	T437N, *de novo*	DEE	FS, Myo	G	Normal	Moderate	DR	Schwarz et al., 2022
12	Male	3 ms	T437N, *de novo*	DEE	FS, Myo, Abs	G	Normal	Moderate	DR	Schwarz et al., 2022
13	Female	2 years	S333T, mc	DEE	GTCS, Myo, Abs	G	Normal	Mild	SF (VPA)	Schwarz et al., 2022

The variants are described using the reference sequence NP_631875.1. General information: EEG, electroencephalogram; F, female; inh, inheritance; M, male; mc, maternal non-affected carrier; MRI, magnetic resonance imaging; ms, months; Mut, mutation; Pat, patient; y, year(s). Diagnosis and seizures: Abs, absences; CSWS, continuous spike and wave during sleep; DD, developmental delay; DEE, developmental and epileptic encephalopathy; EOAE, early-onset absence epilepsy; G, generalized; GTCS, generalized tonic-clonic seizure; ID, intellectual disability; IS, infantile spasms; LGS, Lennox–Gastaut syndrome; Myo, myoclonic; NA, not available; psw, poly-spike wave discharges; SE, status epilepticus; WS, West syndrome. Antiepileptic treatment: CZP, clonazepam; DR, drug-resistant; LTG, lamotrigine; NZP, nitrazepam; SF, Seizure free; VPA, valproic acid.

#### Patient 2

Patient 2 was a girl aged 4 years and 9 months and the first child of a non-consanguineous normal Chinese couple. The patient experienced severely delayed neuromotor development (she was unable to walk independently until 4 years old, with an unsteady gait, and could not understand a single sentence, follow any instructions, or ask), with no ability to speak, and had severe ID (IQ 34). When she was 1 year and 6 months old, the parents found she was subject to spontaneous attacks of eyelid myoclonia (blinking, eyes rolling back to the left, and head moving backward) more than 10 times per day and myoclonic seizures (jerking hands), which usually lasted for 2–3 min and often occurred before sleeping. After the patient was diagnosed with focal onset epilepsy at the local hospital based on an EEG (images were unavailable). on EEG at the local hospital, an antiepileptic treatment was suggested to her. After a poor response to OXC, her seizures were relieved with a combination of LEV/VPA and she was seizure-free last year. At the age of 3 years, the patient came to our hospital, and she was diagnosed with global developmental delay (GDD) with focal epilepsy. EEG showed slowing of the basic occipital rhythm, multifocal generalized complexes of spikes, spike and slow-wave, polyspike and slow-wave, and sharp-wave, significantly at rest and during sleep. Her brain MRI was normal. When the patient was 4 years and 9 months old, upon the last follow-up, the EEG showed the same pattern of slowing of the occipital basic rhythm, multifocal generalized complexes of slow-wave, spike and slow-wave, sharp-slow-wave, and polyspike and slow-wave, especially in occipital, frontal pole, and frontal regions (Figure 1 and Table 1).

### Pathogenic or likely pathogenic variant screening and analysis

**FIGURE 2:**
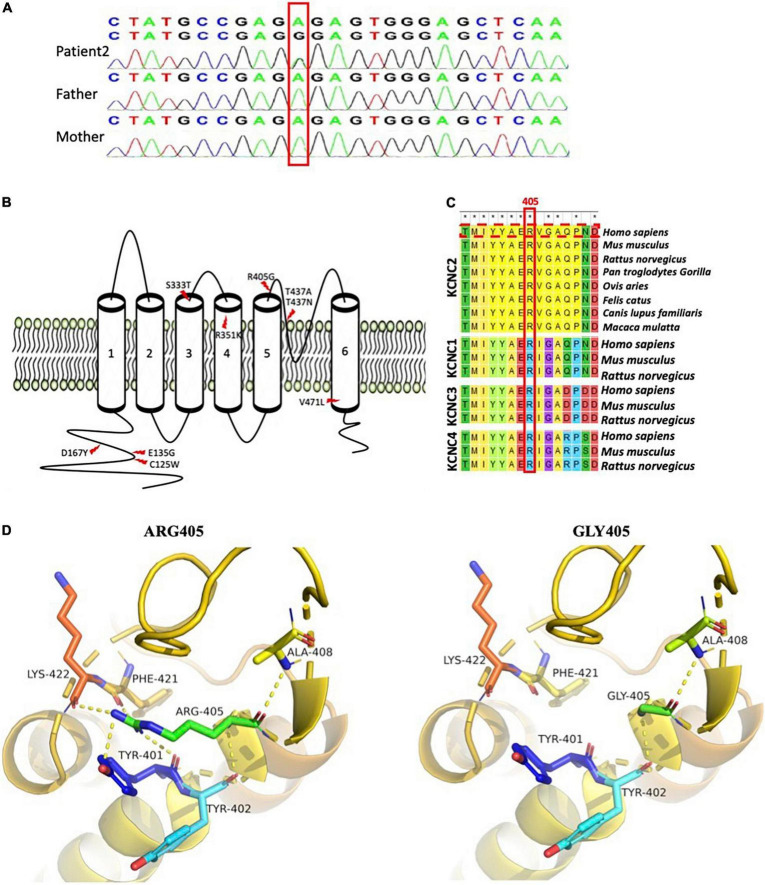
Schematic diagram of known developmental and epileptic encephalopathie (DEE)-linked KCNC2 variants and R405G analysis. **(A)** Sanger sequencing confirms *KCNC2* (NM_139137) c.1213A > G in patient 2 and her parents. **(B)** Nine well-known DEE-linked variants on eight sites of the KCNC2 amino acid chain. Both previously reported R351K and T437N were found in two unrelated patients with DEE, respectively (Schwarz et al., 2022). **(C)** Multiple sequence alignment of KCNC1, KCNC2, KCNC3, and KCNC4 subunits across several species is shown by MEGA7 (the R405 residue and KCNC2 human sequence marked in red boxes). Asterisks (*) denote the highly conserved sites. **(D)** Close view of structural models of the KCNC2 protein (AF-Q96PR1-F1) as ribbons with the p.R405G variant and residues involved in hydrogen-bonding (dotted yellow) network indicated as sticks. (a) Represents wild-type KCNC2 protein with native R405. (b) Represents the mutated KCNC2 with Gly405.

Whole-exome sequencing was performed on both patients. No pathogenic or likely pathogenic variants were found in well-known DEE-associated genes from the OMIM database (OMIM phenotypic series: PS308350). However, we found a heterozygous KCNC2 variant in both patients, NM_139137.4: c.1213A > G (p.R405G). A review of the literature showed that *KCNC2* variants had been documented in several reported DEE cases (Table 1), while R405G had never been documented in public variant or dbSNP. We then performed Sanger sequencing in patient 2 and her parents, which proved the variant was *de novo* (Figure 2A). Unfortunately, further genetic testing was not available for patient 1. Multiple prediction applications, SIFT, PolyPhen2 (HDIV), PolyPhen2, HVAR, Provean, MutationTaster, M-CAP, and REVEL, revealed KCNC2 R405G as damaging or disease-causing. Thus, KCNC2 R405G was likely pathogenic according to the American College of Medical Genetics and Genomics (ACMG) clinical practice guidelines (PS2 + PM2 + PP3). No pathogenic or likely pathogenic variant was found in other genes associated with developmental disorders in both patients.

Based on the reviewed structure data (UniProtKB id: Q96PR1), KCNC2 contains cytosolic N- and C-termini and six membrane-spanning segments. KCNC2 R405 is located within the S5-S6 linker, an extracellular loop that forms one of the ion-selective macromolecular protein pores in KCNC2 (Figure 2B). In addition, R405 is strictly conserved in evolution among multiple species and other members of the K_*V*_3 subfamily, including KCNC1, KCNC3, and KCNC4 (Figure 2C). Analysis of three-dimensional modeling (AlphaFold id: AF-P35670-F1) has revealed that glycine substitution has disrupted the hydrogen bonds that bind R405 with Tyr401, Phe421, and Lys422 (Figure 2D). As a result, R405G is predicted to change the pore structure.

### Electrophysiological characterization

**FIGURE 3:**
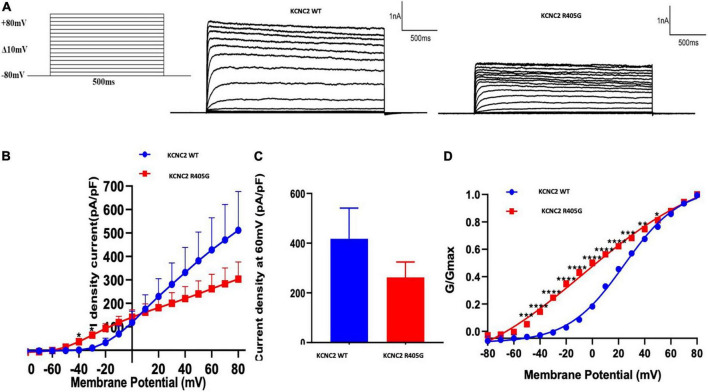
Electrophysiological analysis of wild type and mutant KCNC2 channels. **(A)** Current outward traces from HEK-293 cells transfected with plasmids encoding wild type (WT) or R405G mutant KCNC2 channels in response to the voltage steps from –80 to +80 mV (with an increment of 10 mV). Current scale: 1,000 pA; time scale: 500 ms. **(B)** Averaged current density–voltage curves of WT (*n* = 5) and R405G (*n* = 7) channels. **(C)** Normalized current densities at + 60 mV from HEK-293 cells expressing WT (*n* = 5) or R405 (*n* = 7) channels. **(D)** Normalized conductance versus voltage relationships of WT (*n* = 5) and R405G (*n* = 7) channels. *P < 0.05, **P < 0.01, ***P < 0.001, ****P < 0.0001.

To investigate the impact of R405G in K_*V*_3.2 channel functioning, we constructed EGFP-fusion expression vectors expressing KCNC2 WT and R405G cDNA sequences transfected into HEK-293 cells. Compared to WT, R405G caused a significantly higher density current at voltages between −40 and +10 mV (Figures 3A,B). At +60 mV, the normalized current densities of the channel (262.2 ± 61.91) revealed that R405G caused about 40% less normalized density current compared to the WT on average (262.2 ± 61.91 vs. 427.9 ± 137.1), although the difference was not statistically significant (Figure 3C). By measuring the conductance–voltage curves, we found that R405G shifted the curve to the left, indicating a gain-of-function effect compared to the WT (V1/2 = −6.375 mV, *k* = 49.66 vs. V1/2 = 24.2 mV, *k* = 21.36) (Figure 3D).

### Review of the literature

Seven KCNC2 variants were reported in 11 DEE cases in four published research articles, in which V471L, R351K, and T437N were identified in two unrelated patients, respectively, and a T437A was found in a sporadic DEE case (Figure 2B and Table 1). Six out of seven variants were *de novo*, except S333T in patient 13 (Table 1) was inherited from her unaffected mother, and the authors concluded that S333T was a modifying variant. In addition, none of the seven variants were found in affected individuals without DEE.

## Discussion

The clinical findings in both patients were consistent with the diagnostic criteria for DEE. DEE refers to a group of heterogeneous severe progressive disorders consisting of multiform epilepsies, often intractable, accompanied by developmental delay and ID (Hamdan et al., 2017). In this study, both patients had moderate-to-severe GDD, a well-established indicator for CNS developmental disorders or encephalopathy in early childhood. Consistent with the literature (Rademacher et al., 2020; Vetri et al., 2020; Rydzanicz et al., 2021), we found that focal impaired awareness seizure with an EEG of polymorphic generalized spike and wave complexes and infantile spasms with a typical EEG showing hypsarrhythmia were two traits of seizures attributed to *KCNC2* defects. In addition, no significant brain lesions were found in both patients in this study, which may account for their better response to antileptic drugs (LEV/VPA were provided in both cases) to a certain extent, than patients with significant findings on brain imaging due to *KCNC2-*DEE (Rademacher et al., 2020; Vetri et al., 2020; Rydzanicz et al., 2021; Schwarz et al., 2022; Table 1).

Developmental and epileptic encephalopathy is characterized by high genetic heterogeneity with more than 100 genes associated with DEE or syndromes that meet the definition of a DEE (MIM: PS308350). Among them, synaptopathies and neurological channelopathies are two large groups. DEEs caused by synaptopathies gene variants are usually highly heterogeneous and lack clear genotype–phenotype associations, but neurodevelopmental disorders characterized by central vision impairment due to VAMP2 deficiency constitutes the SNARE complex and hand motor loss in Rett-like syndrome, and myoclonic seizures and subsequent ataxia caused by STX1B pathogenic variants are beneficial to clinical differential diagnosis (Spoto et al., 2022); neurological channelopathies have more related disease types. A typical classification includes epilepsy/migraine, neuromuscular disease, and ataxia, which may be related to the expression-specific organization of ion channel genes (Kullmann, 2010). An example related to specific genotype–phenotype associations is the T-type calcium channelopathy study on *CACNA1H* (Weiss and Zamponi, 2020). Epileptic syndromes caused by CACNA1H defects include a variety of neurodevelopmental disorders and the authors tried to identify the genotypic associations in different phenotypes, including autism spectrum disorder (ASD), immunoglobulin-E (IgE), and neuromuscular disorders. However, the results are not ideal: first, for example, the variant causing ASD may be gain-of-function (GOF) or loss-of-function (LOF). Second, most of the variants listed are only found in a single case. However, a *KCNC2*-associated DEE is not yet well understood. Recently, eight variants, C125W, E135G, D167Y, R351K, T437N, or T437A, V471L, and S333T, are identified in the *KCNC2* gene in DEE cases (Rademacher et al., 2020; Vetri et al., 2020; Rydzanicz et al., 2021; Schwarz et al., 2022), suggesting a subtype of *KCNC2-*DEE. In addition, Rajakulendran et al. (2013) described three affected members in a family who shared the phenotype of cerebellar ataxia, ID, and motor disability. Interestingly, one had epilepsy with abnormal EEG due to a heterozygous 670 kb deletion of chromosome 12q21 that covers full-length *ATXN7L3B* and 3–5 exons of *KCNC2*. Consistently, our findings in two unrelated patients have supported the theory that *KCNC2* is a DEE causative gene.

Identifying the same heterozygous variant in unrelated patients can help investigate disease genotype–phenotype associations. In this study, eight of the nine variants, including R405G, were presumed to be linked to DEE, given that none were found in the general population or patients without DEE. Moreover, we compared the core phenotype of DEE in patients harboring *KCNC2* variants on four sites, R405, T437, R351, and V471, which found amino acid changes in multiple unrelated individuals. The results revealed a remarkable genotype–phenotype association in these cases. In our study, two unrelated patients shared phenotypes, including focal impaired awareness seizure, motor developmental disorder, severe ID, and good response to antiepileptic drugs; in the case of V471, both patients had infantile spasms, severe ID, and ineffective antiepileptic medication (Vetri et al., 2020; Rydzanicz et al., 2021); DEE due to R351K has been associated with significant heterogeneity in the outcomes of encephalopathy. Schwarz et al. (2022) reported two unrelated male infants (7 years and 8 months old); one had severe DD and intractable seizures, while the other one had mild DD and became seizure-free with VPA treatment. However, both patients had generalized tonic-clonic seizures with myoclonic, complex focal seizures, and an EEG characterized by continuous spike and wave during slow-wave sleep (CSWS) (Schwarz et al., 2022). Moreover, three cases were diagnosed with DEE attributed to a variant on T437 in KCNC2, and two patients with the variant T437N had an identical phenotype: febrile seizure and myoclonic epilepsy with generalized EEG features, which exhibited a poor response to antiepileptic drugs and moderate DD, while T437A caused intractable early-onset absence epilepsy (EOAE) with tonic and severe DD (Schwarz et al., 2022). Interestingly, all patients having variants on R405, T437, or R351 had no significant brain lesions compared to both patients harboring V471L, who had widespread hypomyelination and hypertrophic frontal lobes at the age of 8 years (Vetri et al., 2020) or internal and external hydrocephalus at the age of 2 years (Rydzanicz et al., 2021; Table 1). The pathomechanisms of these recurrent KCNC2 variants remain unclear, although findings of the genotype–phenotype associations would be valuable in clinical practice.

KCNC2 R405 is located in a highly conserved K+ selectivity filter region within the ion transport domain, between transmembrane regions S5 and S6. Although high-resolution structures of KCNC2 channels are still unavailable, modeling based on AlphaFold predicted KCNC2 channels revealed that the R405 residue participates in stabilization by forming hydrogen bonds with the side chains of residues in S5 (Y401 and Y402) and loop (A408, F421, and K422), in which three interactions (Y401, F421, and K422) could not be detected in the G405 mutated protein. Thus, the R405G variant would destabilize the channel by impeding such electrostatic interactions. Recently, the changes described for the R405G channel may differ from D167Y and V471L variants associated with DEE. D167Y is close to the N-terminal region (BTB/POZ domain) and may impair the tetramerization of the channel (Long et al., 2005; Rademacher et al., 2020), while V471L impairs the S6 domain function and alters K+ channel gating as the access to the pore (Liu et al., 1997; Labro and Snyders, 2012). Recently, a novel LOF KCNC2 variant V437A was detected in two siblings diagnosed with autism, epileptic encephalopathy, and mild dysmorphism (Mehinovic et al., 2022). However, there are no other cases of association of V437A with autism or DEE.

We performed an *in vitro* electrophysiological analysis, and the results revealed that KCNC2 R405G decreased maximal current density but shifted membrane potential to more negative values in the activation curve of K_*V*_3.2 channels. Currents carried out by R405G mutant were inhibited, exhibiting reduced selectivity for K+ ions, consistent with the location of the R405 residue in the pore helix before the selectivity filter, which interacted with residues in both S5 and pore helices to modulate the selectivity of K_*V*_3.2 channels. During the analysis of the channel kinetics, the R405G mutant displayed a significantly slower deactivation and significantly reduced current compared to WT KCNC2 channels (gain-of-function), which exhibit the same electrophysiological characterization as the D167Y mutant (Rademacher et al., 2020).

In conclusion, we identified a novel *KCNC2* variant and provided additional clinical and genetic findings that suggest a subtype of *KCNC2*-associated DEE syndrome. Comparison of the clinical features in five unrelated DEE cases revealed a consistent genotype–phenotype correlation, while molecular and cellular biology analysis showed that novel variant R405G increased the conductivity at the same voltage, which indicated a gain-of-function alteration due to R405G (Yan et al., 2005).

## Data availability statement

The original contributions presented in this study are publicly available. Information about KCNC2 variant R405G reported in this study can be accessed online using id #0000872152 through the lovd database (https://databases.lovd.nl/shared/variants/0000872152#00010341).

## Ethics statement

The studies involving human participants were reviewed and approved by the Ethics Committee of Wuhan Children’s Hospital, Hubei, China (serial number: 2022497). Written informed consent to participate in this study was provided by the participants’ legal guardian/next of kin. Written informed consent was obtained from the individual(s), and minor(s)’ legal guardian/next of kin, for the publication of any potentially identifiable images or data included in this article.

## Author contributions

SW: designed and conceptualized the study, contributed to the revision of the manuscript, and editing. YY: designed and conceptualized the study. XW: recruited and phenotyped the patients. XD: contributed to the revision of the manuscript. ZL: drafted the manuscript and prepared the figures. JM: conducted electrophysiological studies. WG: conducted molecular analysis and interpreted the data. DS: conceptualization, visualization, resources, supervision, writing – review & editing, and funding acquisition. All authors reviewed the manuscript and agreed to the published version of the manuscript.

## Footnotes

1
https://www.proteinatlas.org/ENSG00000166006-KCNC2/single+cell+type

2
http://chigene.mysxl.cn/#advantages
